# Improving Stylised Working Time Estimates with Time Diary Data: A Multi Study Assessment for the UK

**DOI:** 10.1007/s11205-019-02074-3

**Published:** 2019-02-15

**Authors:** Pierre Walthery, Jonathan Gershuny

**Affiliations:** Centre for Time Use Research, Department of Sociology, University of Oxford, 5 Manor Road, Oxford, OX1 3UQ United Kingdom

**Keywords:** Time use, Working time, Measurement error, Labour Force Survey

## Abstract

Accurate working time estimates represent an important component of the statistical toolbox used for economics forecasting and policy-making. The relatively good availability of such estimates may sometimes induce researchers to take them for granted and see their reliability as largely unproblematic. There is however a growing body of evidence showing that measurement errors may affect their robustness and quality, especially as far as specific but policy relevant subgroups of the population such as part-time or atypical workers are concerned. Against this background, the goal of this paper is to investigate the reliability of paid weekly working-time measurement instruments commonly available in a key UK social survey, the Labour Force Survey. It focuses on the discrepancies between estimates obtained by self-assessed/aggregated instruments—also known as stylised—and those recorded using time diaries which have been found more truthful to the time spent working in ones paid job(s). It is also to explore ways to improve the reliability of stylised estimates in datasets for which no time diary instruments are available, contrasting those where ’usual’ and ’actual’ hours of work are recorded. It does so by creating calibration weights based on the Work Schedule recorded in the 2000 and 2015 UK Time Use Surveys and using them to up/down scale stylised estimates in the 2000 and 2015 UK Labour Force Survey using statistical matching. Such techniques could enable significant improvements of measurement errors in large scale social surveys at a minimal cost.

## Introduction

Accurate working time estimates represent an important component of the statistical toolbox used for economics forecasting and policy-making. Their relatively good availability over the recent decades may sometimes induce researchers to take them for granted and see their reliability as largely unproblematic. There is however a growing body of evidence showing that measurement errors may affect their robustness and quality, especially as far as specific but policy relevant subgroups in the labour force such as part-time or atypical workers are concerned. Against this background, the goal of this paper is to investigate the reliability of paid weekly working-time estimates commonly available in a number of key UK social surveys focusing on the discrepancies between those obtained by self-assessed/aggregated instruments also known as stylised—that is when respondents produce their own estimates—which are the norm in large scale social surveys with those recorded using time diaries. The latter have been shown to be more truthful to the time spent working on ones paid job(s). It is also to explore ways to improve the reliability of stylised estimates in datasets for which no time use instruments are available, by creating calibration weights based on the more accurate diary instrument in a time use survey and use it to up/down scale the stylised estimates conditional to a respondent’s socio-demographic characteristics. Such technique would enable improvement of measurement errors in labour force and employment statistics at a minimal cost.

The paper is divided in three sections. We will first review the limited but consistent literature that has covered this topic before. Using the 2000 and 2015 UK Time Use Surveys (UK TUS) that both contains stylised and time diaries, we will then provide evidence of the extent of the measurement error as well as its likely causes and implications. In a third section, we will examine straightforward pathways for calibrating stylised estimates in the 2000 and 2015 Labour Force Survey using time use estimates from the 2000 and 2015 UK TUS surveys.

## The Quest for Reliable Working Time Estimates

Traditionally, working time measurement in social surveys has been conducted by asking respondents either about the number of hours they spent on paid work worked during a ’typical’ or ’usual’ week or their *actual* hours during a specific reference week, often the week that ended before the interview took place. This should draw our attention to two of the main issues underlying the measurement of paid working time: on the one hand, the definition of ’paid work’ itself, and on the other the nature of the recall effort that is being asked from respondents.

### Defining Paid Work

Beginning with the former, the distinction between ’actual’ and ’usual’ working time found in most social surveys has its source in International Labour Organisation (ILO) resolutions spanning from 1919 to 1994 (Mata-Greenwood 1992). These introduced such concepts as ’normal hours’ which refers to the contracted hours, excluding overtime and ’actual hours’ which encompass any time spent at work or travelling for work, including ’idle’ time, breaks, and preparation for work, but excluding temporary time off work (sick leave, meal breaks).

Further differentiation of actual hours have led to the distinction between productive time (time spent on work duties and tasks) and non productive time (inactive time at work). Both concepts are compatible with the United Nation’s System of National Accounts. It follows that within this framework *usual* working time is defined as the *mode* of the time actually worked over the long period, also including usual overtime (Mata-Greenwood 1992). This official take on paid work does not account for the working-time of those not in employment, which may lead to the overlooking of some forms of casual work.

A closely related notion is that of ’time available for work’ ie time available for one’s employer. It has inspired the definition of actual hours or ’time actually worked’ in a particular job:

(...) all periods, during the reference period, when workers are available to receive orders from an employer or client. This is equal to the time spent performing, or in the course of performing, the tasks and duties defined for that job. (Mata-Greenwood 2001)

Stylised questions in social surveys tend to be influenced by these conceptions, more so if they originate from or are meant to be used by government departments. For instance, in the UK Labour Force Survey, one of the main sources for labour market analysis and forecast which was setup under EU regulation (EC 2014), respondents are asked to provide their hours of paid work on the week that ended the Sunday before the interview, alongside their usual weekly working time, both excluding meal times. In case actual and usual hours differ, respondents have to provide a reason for the discrepancy, for instance whether they work flexible or variable hours. Employees are in addition asked about paid and unpaid overtime and actual hours in their second job. Other surveys instead focus on the more formal definition of contracted hours: for instance respondents to Understanding Society (Institute for Social and Economic Research 2016) are asked about the number of hours they *are expected* to work in a normal week, and average their monthly hours in a second job over a period of 1 month.

Time use surveys by contrast tend to leave it to respondents to label any episode over one or two 24h period—the diary days—as paid work. The Harmonised European Time Use Survey (HETUS) nomenclature, used in the UK TUS allows for some differentiation between commuting, main and second job, breaks, and activities related to paid work. Other time use surveys, such as the American Time Use Survey (ATUS) (United States Bureau of Labor Statistics: American Time Use Survey 2003–2016) offer greater differentiation between work-related activities. In addition instruments such as the Work Schedule which is part of the 2000 and 2015 UK TUS records any 15 min long paid work episode leaving out commuting time, and unpaid breaks over a period of 7 days.

### Measurement Error

The second set of issues affecting time use estimates falls under the broad category of measurement error (Niemi 1993), that is for a given definition of paid work, the extent to which respondents report weekly working time beyond or beneath the ’true’ amount of time spent working during the period under consideration be it the modal value or the actual time spent during the reference week. Measurement error may be large or small and, more importantly, systematic or random, that is associated with one or several respondents’ characteristics. The combination of both characteristics will determine the overall reliability of a working-time estimate.

In most social surveys, respondents are asked to recall and therefore *aggregate*, usually in the space of a few seconds the time they actually worked last week and/or the modal value of their working time over period of time left to their discretion, both of which involve complex cognitive tasks. The ability or willingness of respondents to report accurately on a behaviour such as paid work should not be taken for granted (Schwarz and Oyserman 2001; Sonnenberg et al. 2012). Another well known source of measurement error is social desirability which, it has been argued, affects the recall and in some cases even the actual behaviour of survey respondents. A typical example is ’Busyness’ being seen as a ’badge of honour’ among certain groups of service workers (Gershuny 2005). It is also thought that the more specific and the (temporally) closer the behaviour of interest from the interview, the more accurate the estimate (Galtung 1970) in Niemi (1993).

Although arguably cheap and convenient to administer, stylised questions are by no means the only way to measure working time. A limited but consistent tradition has attempted to empirically assess working-time using alternative methods such as experiential sampling (where individuals are randomly prompted by a device to report whether they are working or not) or time diaries. As a result a number of studies have attempted to assess the relative accuracy of these methods over each other (Juster et al. 2003). Time use diaries have been usually deemed more reliable and robust to respondent bias and than stylised questionnaires (Carp and Carp 1981; Robinson 1985; Sonnenberg et al. 2012). An indication that measurement error might be an issue with stylised estimates is that compared with other methods the standard errors of the estimate they provide tend to be larger (Kan and Pudney 2008). Such robustness test have been done in the field of housework (Kitterød and Lyngstad 2005; Kan and Pudney 2008; Schulz and Grunow 2012), whilst less scrutiny has been directed towards paid work estimates.

#### The Nature of the Gap

Several authors have reported that overall stylised estimates tend to overestimate working hours as reported in time diaries. This phenomenon was first noted by official statisticians of the labour market in the 1980s (Hoffmann 1981; Niemi 1993), and evidence has gradually accumulated over the subsequent decades. Robinson and Gershuny found a consistent gap of this sort for employed people in eleven Western countries for which data was then available (Robinson and Gershuny 1994). Subsequent evidence accumulating from the Multinational Time-Use Study (MTUS) shows this phenomenon consistently in various European and North American countries wherever appropriate data has been collected, increasing in size since the earliest observations in 1965. Empirical studies have also shown that ’actual’ hours of work tend to be closer to diary estimates than ’usual’ ones (Frazis and Stewart 2004). Stylised question tend to perform best when activities are externally defined, happened on a regular basis and worst in case of sporadic short term activities (Sonnenberg et al. 2012).

The same authors observed that this gap substantially increased in size as the stylized estimates grows beyond 40 hours per week. This has led to claims of a generalised overestimation of working-time by stylised estimates (Robinson 1985; Robinson et al. 2011) in traditional surveys sometimes accompanied by additional claims of a long term decline in working hours in Western societies. This has been conflicting with other reports documenting the rise of overworked employees (Jacobs 1998) in the Unites Stated and triggered counter claims that gaps between time diary and stylised estimates are in fact random variation or ’regression to the mean’ moving around a ’true’ population value.

Other critics have stressed that Robinson’s original results were based on synthetic (i.e. reconstructed weeks) rather than actual weekly data of individual respondents, the US Current Population Survey only collect diary data for 1 day, and therefore may suffer from its own measurement bias (Frazis and Stewart 2004). The same authors found that whilst an overestimation of about 5% was found between the two, when comparing stylised actual rather usual hours, that is hours worked last week, sizeably smaller discrepancies could be observed. It also found that whilst those working long hours tended to overestimate their working-time, those working under 20 h tended to overestimate them (Frazis and Stewart 2014). Since the latter group is larger in size, this may account for the overall positive gap between diary and stylised estimates. These results meet another concern, more sociological in nature: appearing to work long hours may not be socially desirable for everyone in the population. Robinson et al. (2011) subsequently restated their claims, using more recent data. Part of the differences might be due to the fact that not all of those who are in paid work —the population considered by Robinson —do actually report some work, which may have a downward effect on the means of hour worked (Frazis and Stewart 2014).

#### Correlates of the Gap

Further empirical studies have confirmed that there is not a single explanation for measurement error in stylised working-time questions. The gaps between stylised and time diary estimates of paid working time whether under-or over-estimations of the latter by the former have been found associated with gender, reported hours of paid work and employment status (Bonke 2005; Frazis and Stewart 2014). To start with, the gaps vary systematically according to the duration of the respondents working schedules. Workers who report working between 35 and 45 stylised weekly hours have their time diary estimates come close to the former. Greater gaps emerge for people reporting longer work days and weeks (Robinson and Bostrom 1994; Robinson and Gershuny 1994), with workers estimating 60–80 h work weeks showing the greatest gap, suggesting a tendency of stylized time estimates to follow a ’the higher the estimate, the greater the overestimate’ pattern. Bonke (2005) as well as Frazis and Stewart (2014) have shown that there is an inverse relationship between the number of hours worked and the nature of the gap: stylised estimates tend to be lower than the time diary estimated ones when hours of works drop below 30 hours per week, and higher when they rise above 45 h. Similar results were shown by Robinson et al. (2011), using Belgian and US data, but not analysed systematically. Consequently also, the gap between stylised and time diary estimates of working time tends to narrow when full-time employees only are selected (Juster et al. 2003).

Whilst there is agreement that gender and measurement error are associated, conflicting accounts have emerged about its precise nature. Recent research has claimed that women are less accurate than men and tend to over estimate their paid working time (Bonke 2005), which is attributed to the fact that women tend to have more complex daily schedules than men, having to reconcile between greater amount of paid and unpaid work. On the other hand, on four occasions (1965, 1976, 1995,1999) women were found to repeatedly underestimate their working time in the USA (Juster et al. 2003). Robinson et al. (2011) using recent Belgian data, also found that women working more than 50 hours per week, tended to overestimate their working time to a greater extent then men.

A further aspect that to our knowledge hasn’t been explored is the relationship between measurement error and other characteristics of paid work, likely to be related. Based on the empirical studies mentioned above, it would also be natural to expect a relationship between regularity of working time and accuracy: the more irregularity in a respondent’s work schedule, the more likely s/he will be of experiencing recall errors. For similar reasons, unsocial hours, that is hours of work outside the 8AM–6PM window or at the weekend should also be associated with more imprecise estimates, given that they would interfere with respondent other areas of social life. For the same reason, self-employment should be expected to yield more error, due to the absence of predictable schedule it entails, although it could be argued that by virtue of being hourly paid, respondents should more readily produce accurate estimates of their working time.

*Types of time use surveys* The majority of time use surveys is limited to 2 days diaries, in order to minimise respondent burden. Respondents are usually asked to provide a detailed account of their activities at 10 min intervals over two distinct 24 h periods, one during the working week, the other one at the weekend. The American Time Use Survey (ATUS) is an exception as it relies on a single day diary. The main downside is that person-level weekly working-time cannot directly be estimated from the data. Therefore a ’synthetic week’ has to be constructed by separately computing the average working time for each day of the week, or assumptions made about the number of days worked during the week for each respondents. A recent development has consisted in using week long time diaries recording a limited number of activities, typically paid work, also called ’work-grid’ or ’work schedule’ allowing researchers to directly compute weekly working-time estimates by aggregating 7 days at the individual level. Such diaries have been pioneered in France, Belgium and the UK (Robinson et al. 2002, 2011). Both in the 2000 and 2015 UK Time Use Surveys respondents filled in such a week-long work schedule (WS) in which any paid work excluding lunch breaks and commute was reported at intervals of 15 min.

*Calibration* Time diaries aren’t routinely included in social surveys as they are costly to administrate and add to respondent burden. As a result, the suggestion to use separate time use surveys to calibrate stylised estimates in traditional surveys has come to prominence in several substantive areas (Kan and Gershuny 2009; Borra et al. 2013). A possible approach consist in modelling the gap between stylised and time diary estimate in a survey which includes both types of measurements, then use the regression coefficients to compute calibration indices or weights matching key characteristics of respondents (Kan and Gershuny 2009). A variant of the same approach would consist in directly modelling stylised estimates using the diary value as a co-variate (Gershuny et al 2005). Once computed, average values of the calibration indices can be matched for a number of subgroups in a fashion similar to post-stratification weights. So far this has been achieved for daily working time only, and calibration was computed using a survey with a relatively small sample size, Home on Line (Kan and Gershuny 2009). Propensity score matching has also been used to directly match time-diary derived working time for individuals sharing similar characteristics, thus skipping the modelling stage, an approach that has been deemed preferable due to the fact that it does not freeze the variability of time-use measurement (Borra et al. 2013). To our knowledge, no assessment of calibration techniques of weekly working time estimates has been made in the UK. We aren’t aware either of any attempt at calibrating mainstream social survey such as the Labour Force Survey using time use estimates.

*Summary* Stylised estimates are prone to systematic measurement errors, although the extent of these errors as well as the characteristics with which they are associated is still under debate. There is however a consensus that the overall duration of working-time as well as gender matter for measurement error, whilst it is still unknown whether and how variations in the daily schedules, working unsocial hours, being self employed impact on it to the same extent. Calibration indices may be computed based on these covariates to improve stylised estimates, and in a small number of cases —unrelated to paid work —have been implemented. Discussions have been taking place about the optimal method with which calibration weights should be applied to traditional datasets that do not include time use variables, either by simply aggregating means according to the marginal distribution of a number of relevant characteristics or using more advanced techniques such as propensity score matching. The contribution this paper is making is therefore to provide further empirical evidence of the nature and extent of the gap between stylised and time-diary estimates of weekly working time in the UK, using data from 2015 and a more robust approach than previously as it relies on a weekly time use instrument. It is also to compute calibration weights in order to improve the observed gap.

## Data and Methods

This study was conducted in three stages. A descriptive analysis of the differences between the stylised (both usual and actual hours) and time diary (i.e. work schedule) estimates of weekly working time was first conducted by gender, duration and variability using the 2000 and 2015 UK Time Use Surveys. The second stage consisted in modelling the difference between stylised working time and the work schedule. In the third stage the previously predicted values were imported into the UK Labour Force Survey,which only recorded stylised working time. A comparison of calibrated and uncalibrated working-time estimate was then conducted.

Four surveys were used for this study. The UK Time Use Studies (UK TUS) from 2000 and 2015 were the primary sources for time use data. The 2000 and 2015 UK TUS are random household surveys of UK residents aged 8 and over. Sample size in 2000 was 11,664 individual respondents in 6414 households who completed 19,898 diaries and 6131 week-long work schedules.^1^ In 2015, 9,388 respondents in 4,238 households completed 16,550 diaries and 3,523 work schedules. Of these 4375 and 2986 working age respondents in employment completed the work schedule respectively in 2000 and 2015. These provided information about the time spent in paid work excluding lunch breaks and commute at 15 min intervals over a period of 7 days. Both surveys also included stylised measurements of working time: in the 2000 UK TUS respondents were asked about their usual hours of work in their main and second jobs as well as paid and unpaid overtime. In the 2015 UK TUS respondents were asked to provide their usual and actual working time. The question was asked separately to employees and self employed. Employees were also asked about the number of hours they spent on actual paid overtime as well as their working time in all of their other businesses/jobs rather than specifically their second job, as it is assumed that the self-employed do not work for a number of hours that is fixed contractually. Although they both exclude meal breaks, both definitions therefore are not perfectly identical. In the 2015 TUS, the self-employed were not asked about whether they worked part-time: as a result this was derived from their usual hours of work instead. Actual hours, that is hours worked last week were not recorded in the 2000 TUS, and usual hours are only asked about the respondents’ main job in the LFS. As a results it is only in the 2015 TUS that both estimates are strictly comparable.

For the calibration stage, annualised versions of the UK Labour Force survey were produced by combining four quarterly datasets matching the period during which fieldwork respectively the 2000 and 2015 UK TUS were conducted and removing redundant observations. The LFS is a stratified random household survey of UK residents taking place on a continuous basis. Yearly sample size was 39,616 working age respondents in 2000 and 35,192 in 2015.

*Modelling and calibration*

The first stage of the calibration of usual and actual working-time estimates in the LFS relied on OLS modelling of the difference between respectively stylised weekly hours of work and respondents’ more precise time diary (i.e. work schedule) records in both the 2000 and 2015 TUS. A statistical matching procedure was applied through which individual records of the UK-TUS were added to similar respondents in the LFS, these were then used to calibrate stylised weekly working time estimates in the LFS. For each year and instrument type (i.e. usual or actual) two models were tested.

In Model 1 usual and actual hours are regressed against *daily* working-time and its coefficient of variation (CV) in order to account for the impact of the duration of the working day and its predictability on recollection, with the assumption that the greater the variation over the week, the more complex the cognitive tasks required to aggregate them into an estimate of weekly working time. By contrast with the more widely used standard deviation, the value of the coefficient of variation is not directly associated with the quantity that is measured, as it is expressed as a proportion of the mean. This variable was tested both as a main effect and in interaction with *daily* working time. The number of days worked as well as the proportion of the time working unsocial hours (before 8 AM, after 7PM or at the weekend) were also included in Model 1.

1 $$\begin{aligned} d_s=\beta _0 + \beta _1t_d + \beta _2 CV(t_d)+ \beta _3n_d + \beta _4p_c+\beta _5t_d*CV(t_d) +\varepsilon \end{aligned}$$

with:

- $d_s$ the difference between the usual or actual weekly working time and the WS-recorded working time.
- $t_d$ the average number of minutes worked per day
- $CV(t_d)$ the coefficient of weekly variation in the number of minutes worked per day
- $n_d$ the number of days worked during the week
- $p_c$ the percentage of weekly working time spent working unsocial hours
- $t_d*CV(t_d)$ an interaction term between the weekly number of days worked and the CV of daily working-time.

In a Model 2, a vector of socio-demographic variables $X_k$ were added:

2 $$\begin{aligned} d_s=\beta _0 + \beta _1t_d+ \beta _2 CV(t_d)+\beta _3n_d + \beta _4p_c+\beta _5t_d*CV(t_d) + \beta _kx_k+\varepsilon \end{aligned}$$

These accounted for occupation,employment status (i.e. self-reported part-versus full-time, self-employed), whether respondent held a second job, gender, age, age squared, partnership status, age of the youngest child and education. A three way interaction term was added between part-time employment, gender, and self-employment.

Once the predicted values of the differences in weekly hours were obtained, ’naive’ matching was initially carried out by computing averages of $\hat{d_s}$ by year, gender, and 5-h bands of the original actual or usual weekly working time and matching them to the respective values in the Labour Force Survey datasets. Results proved disappointing, and therefore a more systematic approach, based on statistical matching was adopted: micro (i.e. observation-level) exact non parametric matching was performed using a nearest neighbour algorithm. (Borra et al. 2013; D’Orazio et al. 2017; D’Orazio 2017). Matching distances were computed using the same set of socio-demographic variables $x_k$ that were included in computing the model described above. All analyses were carried out using R (2018).

## Results

**Table 1:** Description of the datasets

	LFS		TUS	
	2000	2015	2000	2015
Weekly working hours (usual)	38.8	37.8	38.3	36.8
Weekly working hours (last week)	37.0	36.6	38.4	37.4
Weekly working-time (work schedule)	–	–	35.8	36
*Weekly working time (banded)*	–			
1–30 h	25.8	25.1	25	27.6
31–45 h	46.6	51	43.8	46.9
46+ h	27.6	23.9	31.1	25.6
CV Daily working time	–	–	.88	.97
Weekly % unsocial hours worked	–	–	18.3	18.2
Number of days worked	–	–	4.7	4.3
Daily working minutes (work schedule)	–	–	307	312
Female	46.4	47.9	48.7	48.8
Age	39.4	41.3	39	42.8
Self-employed	11.9	13.9	11	15.1
*Education*	–			
Degree	16.2	30.3	17.2	37.8
Higher	9.3	9.3	12.3	13.2
Secondary or lower	74.5	60.5	70.5	49
*Occupation*	–			
Managers	16.4	10.4	15.7	14.4
Professionals	10.2	19.1	12.5	15.6
Assoc. professionals	10.3	13.5	10.9	17.1
Administrative	15	11.1	15	10.5
Skilled trades	12.1	11.3	12.7	10.9
Caring and leisure	10.8	9.4	6.5	9
Sales and cust services	8.5	7.8	6.9	6
Machine operatives	9	6.7	7.2	7.4
Elementary	7.8	10.8	12.5	9.2
*Age of the youngest child (banded)*	–			
0–4 years	–	–	14.1	16
5–9 years	–	–	12.4	9.8
10–17 years	–	–	16	17.4
*Partnership status*	–			
Single, never married	–	–	11	19.9
Married/cohabitating	–	–	81.8	70.3
Divorced/widowed	–	–	7.3	9.8

All respondents aged 16-64 in employment. data: UK TUS 2000 and 2015; Annualised UK LFS 2000 and 2015

An initial examination of the two most recent large scale time use surveys available in the UK, as well as the LFS data for the same years show a consistent distribution of the variables of interest (Table 1). The 2000 LFS sample has slightly fewer female respondents than the other datasets. Respondents in the 2015 UK TUS were also 1 year older on average than in the LFS. An hour and a half difference in the usual weekly hours is noticeable between the two time use surveys, which is close to the drop in usual weekly working time between the 2000 and 2015 LFS. By contrast, weekly working time according to the work schedule remained stable at about 36h per week.

**Fig. 1:**
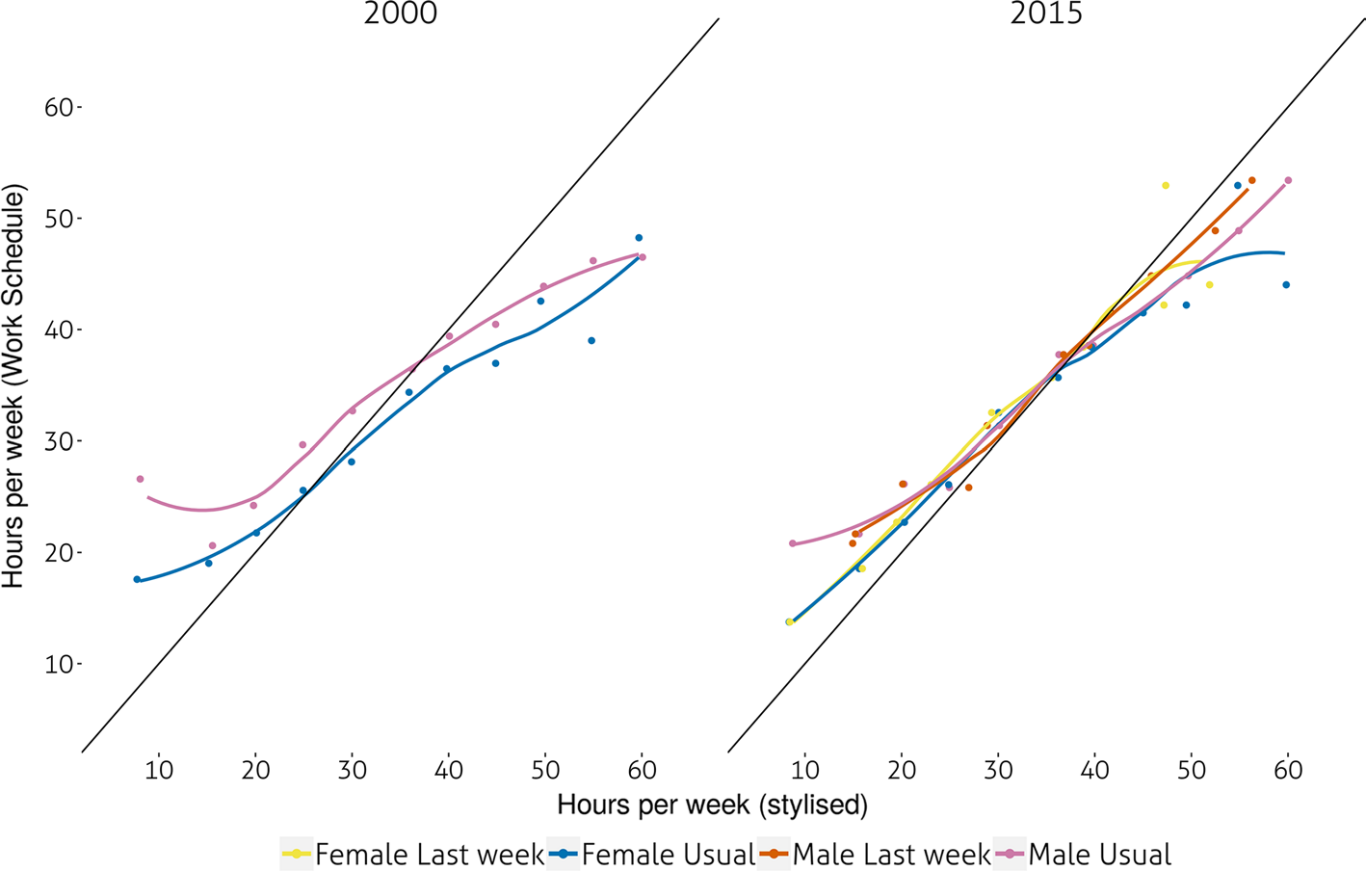
Gaps in estimated weekly hours of work by gender and stylised instrument. Mean weekly working time (work-schedule) versus mean actual/usual weekly working-time by 5 h intervals of mean WS estimates and gender. All respondents aged 16–64 in employment. Data: UK TUS 2000 and 2015

Let us now examine the extent of the gap between stylised and Work Schedule (WS) estimates of weekly working time. Figure 1 shows ’equality’ plots which follow a logic similar to those found in Gershuny et al (2005). First, both stylised and work schedule observations are grouped into 5 hour bands, beginning and ending respectively 2.5 h below and above each multiple of 5 h ranging from 5 to 70. For all respondents falling into these bands the mean of the original number of hours (i.e. usual and/or actual and work schedule) are computed and then plotted against each other. For instance, in Fig. 1 men who claimed working usually around 20 hours per week recorded an average of 23 hours in the work schedule, an indication that they might have been underestimating their stylised hours. A ’line of equality’ shows where dots would be located if both stylised and work schedule estimates were identical.

There are indications that in 2000, men usually working less than 35 h and women working less than 25 h underestimated their working time in the stylised instrument whilst those working more than 40 h overestimated it. Conversely, for the majority of those working between 30 and 40 h there was little difference between stylised estimates and the work schedule. Women and men working between 30 and 40 h per week are those providing the most reliable estimates of their weekly working time. Figure 1 also shows that an asymmetrical gender divide is present: male respondents who worked less than 35 hours per week tended to underestimate their working time to a much larger extent than women. At the same time, men who worked more than 45 hours per week tended to overestimate their working time, less so than women. In other words, it appears that men working short hours and women long hours are the worst ’culprits’ in terms of misreporting their working time in stylised questionnaires. A third result is that respondents reporting their actual hours in 2015, especially women, tended to be more accurate than when reporting their usual working time. This may also show that actual hours tend to be more accurately aggregated by respondents than usual ones. Unfortunately, we cannot carry out the same comparison for 2000 as only stylised usual hours were recorded then. It should also be pointed out that there were far more respondents reporting hours below 30 than above 40.

**Fig. 2:**
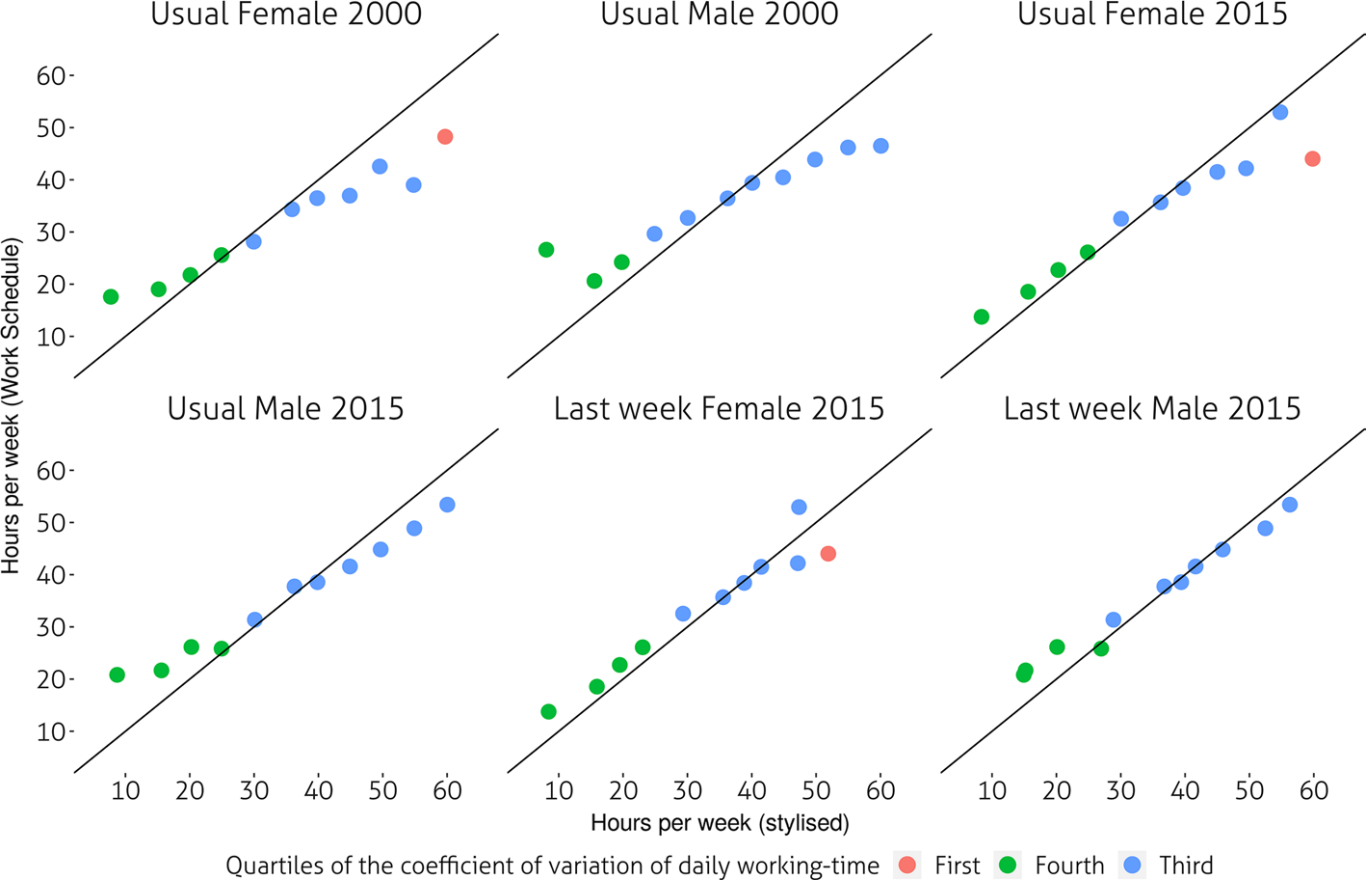
Gaps in estimates of weekly hours of work by variations in daily working-time. Mean weekly working time (work-schedule) versus mean actual/usual weekly working-time by 5 h intervals of mean WS estimates and quartiles of the coefficient of variation of daily working time over 7 days. All respondents aged 16–64 in employment. Data: UK TUS 2000 and 2015

Figure 2 provides some limited evidence of association between variations in the duration of the working day throughout the week and the size of the gap between WS and stylised hours. It is mostly among women working long hours and whose *usual* daily working time tend to vary (third quartile of the coefficient of variation of daily working-time) that stylised and work schedules estimates diverge the most. This is true of both 2000 and 2015 respondents. However, neither women nor men reporting their actual hours showed the same trends. A comparable result, although to a lesser extent can be found among respondents whose daily working time tend to vary the most and who work less than 20 hours per week (Fourth quartile of the coefficient of variation of daily working time).

**Fig. 3:**
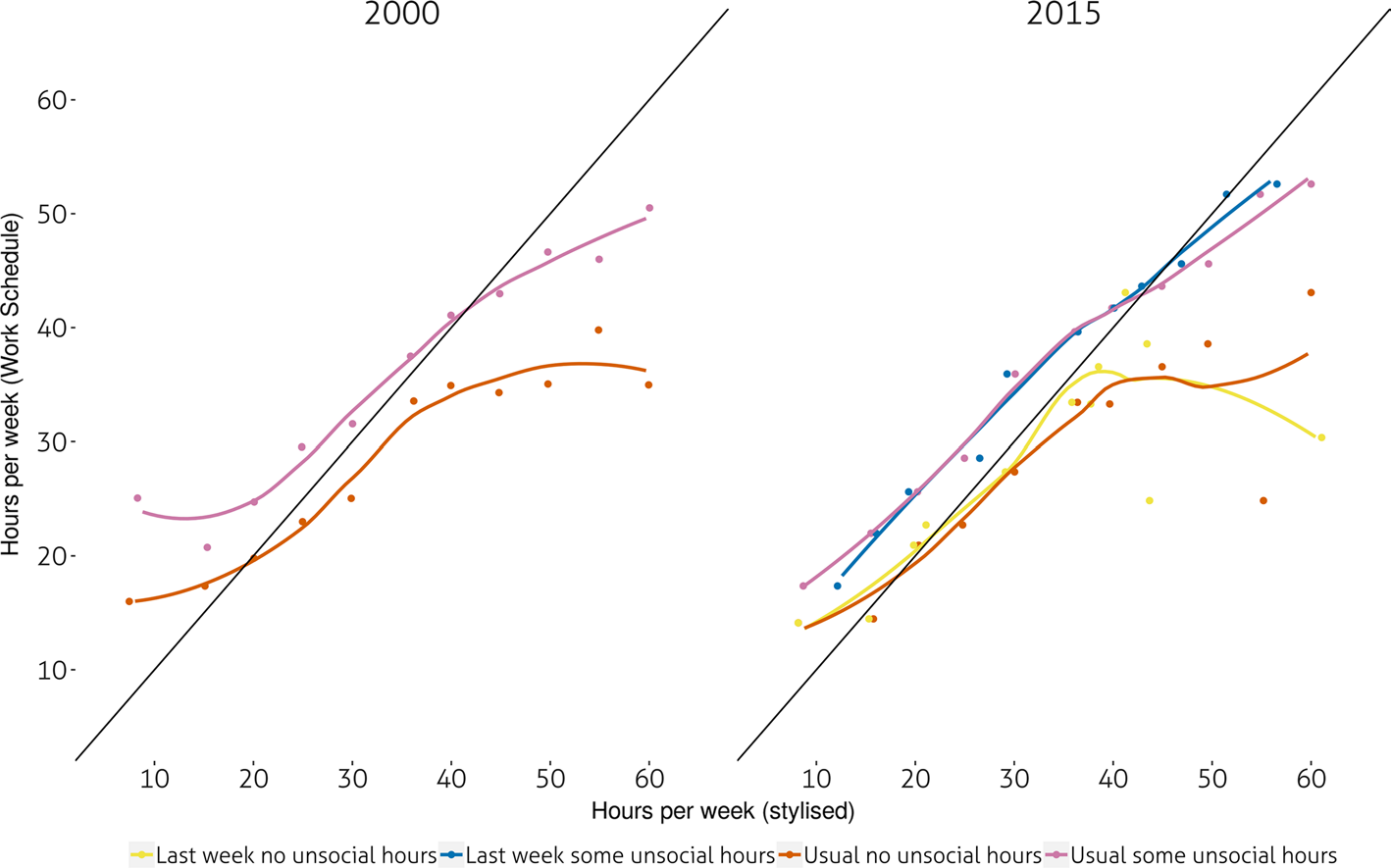
Gaps in estimates of weekly hours of work by schedule type. Mean weekly working time (work-schedule) versus mean actual/usual weekly working-time by 5 h intervals of mean WS estimates and whether respondents work unsocial hours (before 8 AM, after 6 PM weekdays, at the weekend). All respondents aged 16–64 in employment. Data: UK TUS 2000 and 2015

Finally, Fig. 3 shows that whether respondents usually worked unsocial hours (defined as working either at the weekend, before 8AM or after 6PM) had an impact on the gap between stylised working time and work schedule. Those who worked such unsocial schedules tended to *underestimate * their hours both in 2000 and 2015, irrespective of whether these were usual or actual. At the same time, those *not* on unsocial schedules tended to vastly overestimate their working-time, especially if they claimed working above 40 hours per week in the stylised instrument. Additional bivariate analysis (not shown) hinted that the highly educated were slightly more likely to underestimate their hours. No bivariate association with age was found.

### Calibrating Labour Force Survey Estimates

We now move to computing calibration scores in order to correct for the patterns of over-and underestimation just described. Initial trials showed that modelling the gap itself (Bonke 2005) rather than stylised hours directly (Kan and Gershuny 2009) provided better results.

We proceeded in two stages. First, we computed OLS models of the gap between respectively usual (2000 UKTUS) and usual and actual working time (2015 UKTUS) and the WS. The model-predicted differences were then imported into annualised 2000 and 2015 LFS datasets.

As visible in Table 2, the best fitting models for both 2000 and 2015 data are those that simultaneously account for the daily and weekly variability of working time and unsocial schedules but also socio-demographic controls with a R$^2$ value higher than $>.5$ (Models 2, 4 and 6). Given the variables included, the coefficients are likely to be inflated due to collinearity, therefore some caution is needed when interpreting these results. Predicted values, which are our main interest here, should remain unaffected. As expected, the coefficient of variation of the daily working time in other words, the variability of daily working time over 7 days does predict the gap between stylised and WS hours albeit to a declining extent as further covariates were included in the model. The same is true of daily working time and the number of days worked. Having two jobs and being self-employed both had significant positive impact on the size of the gap.

Although most variables show comparable patterns of association in 2000 and 2015, a number of differences stand out. The coefficient of variation of daily working time is *positively* related to the gap between usual and WS hours in 2000, whereas the association is *negative* in 2015 for both usual and actual hours, once socio-demographic controls have been taken into account. This also applies to the number of days worked and the interaction between being female and having a second job. For 2015 data, where both actual and usual hours were recorded, the fit of the model was better for the usual hours, with a R$^2$ value of $=.67$, against $=.57$ for actual hours.

**Table 2:** OLS regression output of stylised weekly working time, UK TUS 2000-15

	2000		2015			
	Usual		Usual		Last week	
	Variance	Full	Variance	Full	Variance	Full
Intercept	− 16.51 (3.39)***	7.95 (3.53)*	22.72 (2.62)***	43.72 (3.03)***	22.14 (2.92)***	39.39 (3.65)***
CV (daily hours WS)	13.04 (1.22)***	7.66 (1.02)***	1.55 (.96)	− 3.98 (.78)***	2.17 (1.07)*	− 2.94 (.95)**
Days worked	3.03 (.51)***	2.06 (.43)***	− 2.98 (.39)***	− 4.13 (.31)***	− 3.39 (.43)***	− 4.51 (.38)***
Weekly % unsocial hours	− .04 (.01)***	.01 (.01)*	− .02 (.01)**	.02 (.01)*	− .01 (.01)	.03 (.01)***
Daily working time	− .05 (.00)***	− .09 (.00)***	− .04 (.00)***	− .07 (.00)***	− .03 (.00)***	− .06 (.00)***
CV(DWT) * WS hours	.04 (.00)***	.02 (.00)***	.01 (.00)**	− .00 (.00)	.01 (.00)**	− .00 (.00)
Female		− 3.93 (.45)***		− 2.28 (.42)***		− 1.92 (.51)***
Age		.51 (.10)***		.38 (.11)***		.41 (.13)**
Age squared		− .01 (.00)***		− .00 (.00)***		− .00 (.00)**
No children	–	–	–	–	–	–
Youngest child is 0–4		− .44 (.54)		− 1.22 (.51)*		− .04 (.63)
Youngest child is 5–9		− 1.39 (.56)*		− 1.55 (.59)**		− 1.95 (.73)**
Youngest child is 10–17		− .42 (.49)		− .36 (.46)		.38 (.56)
Part-time		− 18.69 (1.01)***		− 16.45 (.87)***		− 16.19 (1.01)***
Degree or above	–	–	–	–	–	–
Above GCSE		− .81 (.54)		− .07 (.42)		− .46 (.51)
GCSE and equ.		− 1.75 (.57)**		− .39 (.55)		− .43 (.67)
Below GCSE		− 1.68 (.72)*		.01 (.72)		− .56 (.90)
Other		.05 (1.06)		− .85 (.71)		− 1.65 (.86)
Managers	–	–	–	–	–	–
Profess.		− 2.16 (.67)**		− .72 (.60)		.27 (.73)
Assoc. prof.		− 4.49 (.66)***		− 2.93 (.58)***		− 1.93 (.71)**
Admin.		− 4.34 (.63)***		− 2.86 (.66)***		− 2.01 (.82)*
Skilled		− 3.19 (.66)***		− 1.14 (.69)		− .05 (.84)
Caring/Leis		− 4.55 (.81)***		− 3.39 (.71)***		− 2.66 (.88)**
Sales/cust serv.		− 4.97 (.82)***		− 3.09 (.80)***		− 1.80 (.98)
Mach. oper.		− 1.49 (.79)		− 1.50 (.77)		− 1.10 (.94)
Elementary		− 5.35 (.70)***		− 3.25 (.73)***		− 1.52 (.87)
Single, never married	–	–	–	–	–	–
Married/cohabitating		.64 (.56)		.17 (.49)		.42 (.58)
Divorced/widowed		.01 (.82)		.31 (.71)		.36 (.86)
Has a second job		8.58 (.72)***		9.12 (.73)***		9.39 (.91)***
Self-employed		1.99 (.69)**		5.16 (.64)***		3.38 (.77)***
Female and part-time		4.23 (1.07)***		2.68 (.93)**		3.34 (1.10)**
Female and self-employed		− 3.72 (1.40)**		− 2.40 (1.12)*		− 4.26 (1.36)**
Part-time and self-employed		.73 (1.60)		− 3.63 (1.20)**		− 1.90 (1.46)
Adj. R$^2$	.28	.51	.47	.67	.43	.57
Num. obs.	3895	3895	2749	2749	2749	2749

$$***$$$$p<.001$$; $$**$$$$p<.01$$; $$*$$$$p<.05$$. Respondents aged 16–64 in paid work. data: UK TUS 2000 and 2015

**Fig. 4:**
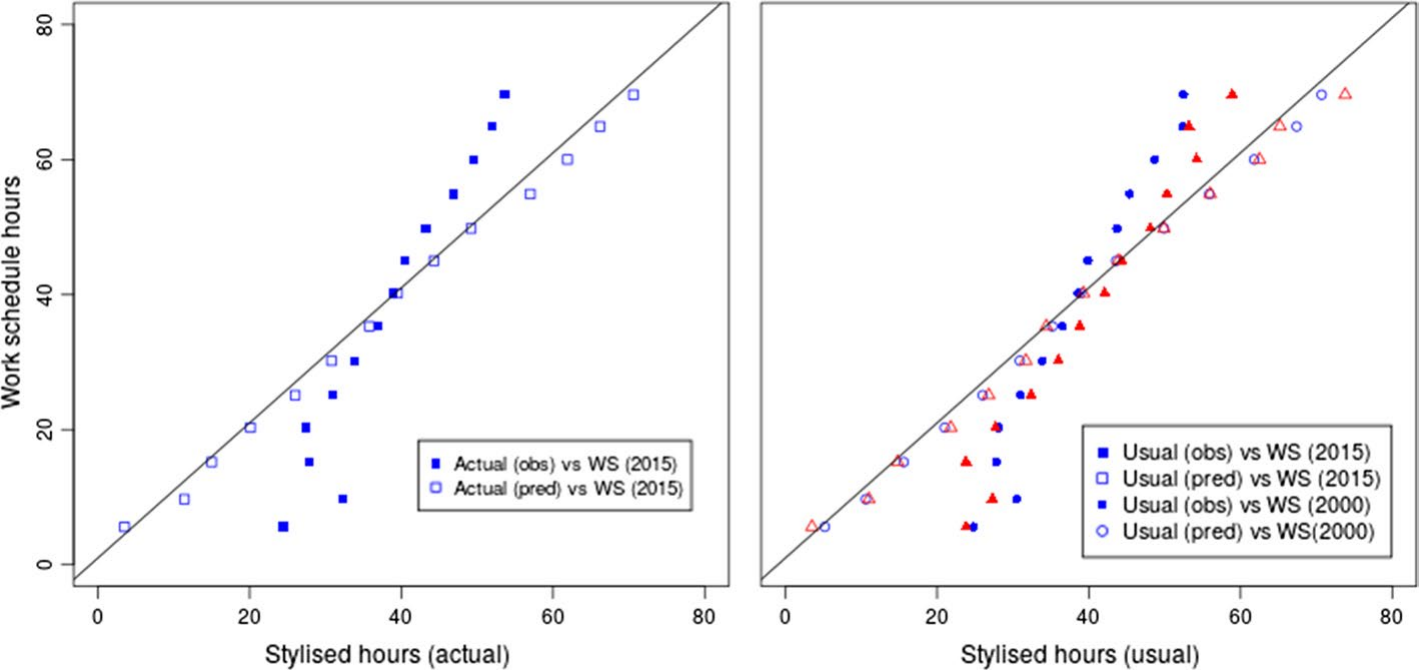
Stylised versus regression predicted estimates, TUS

In order to compare the quality of the values predicted by the model, we first used them to calibrate the stylised hours originally recorded by the respondents in the 2000 and 2015 TUS and plotted them against the results from the work schedule. As can be seen in Fig. 4, in both 2000 and 2015, the stylised hours calibrated with the predicted values of the models adequately correct the gap, as they come close to those from the estimates recorded with the work schedule. The models seem equally good at correcting the gap between work schedule and stylised estimates in 2015 and in 2000. These results are markedly better than in a model where the stylised hours were directly used as dependent variable (not shown).

**Fig. 5:**
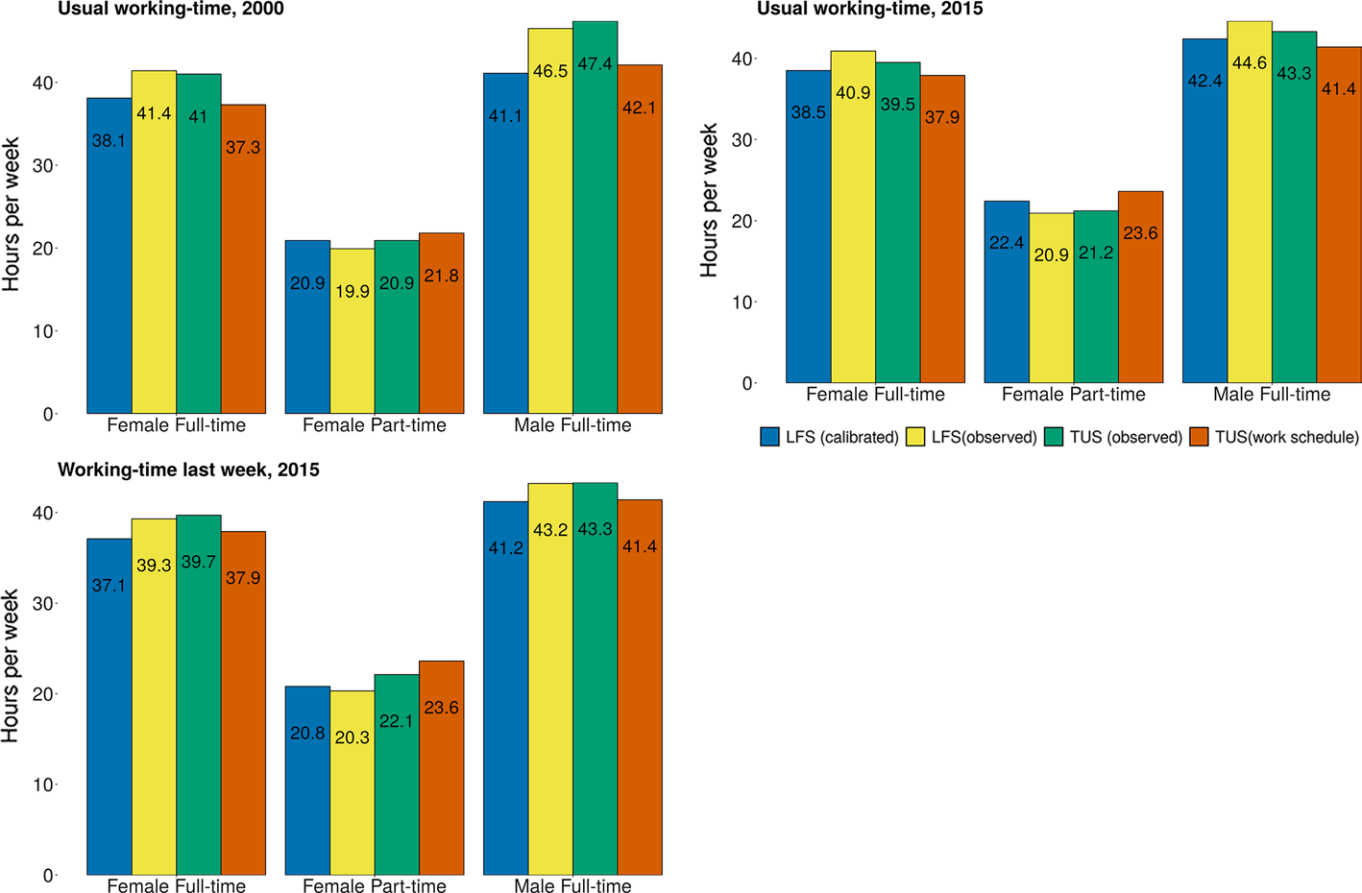
Observed vs calibrated working-time estimates, usual and actual working-time. Mean weekly usual, usual calibrated and work-schedule recorded weekly working time by gender and employment status. All respondents aged 16–64 in employment. Data: UK TUS 2000 and 2015; UK LFS, Annualised dataset 2000 and 2015

We can now turn to the calibrated LFS results. The large size difference between the LFS and the TUS meant that the same observations from the TUS will be matched several times to different observations in the LFS. The resulting dataset, in effect retaining all observations from the LFS whilst including the predicted values from the TUS models described above was obtained from the statistical matching. Since we cannot directly plot work schedule vs calibrated values in the LFS dataset, we opted instead for comparing mean working time among full-time and part-time employees by gender. Figure 5 presents the results contrasting results in the TUS with those in the LFS.

The results in Fig. 5 show that calibration clearly improve LFS estimates. On the one hand, in all cases, the original stylised LFS estimates were brought closer to the those calculated with the work schedule in the TUS. This was achieved by decreasing the value of the mean working time estimates for part-timers, and increasing it for full-timers. In six cases out of nine as visible in Fig. 5, the differences between work schedule and calibrated LFS estimates fell to 1 h or below, down from significantly higher gaps. Calibrated estimates performed particularly well with respondents working full-time. In one case the aggregate difference was reduced to about 20 min for the actual hours of male respondents working full-time in 2015, down from an initial gap of about 2 h. Calibrated part-time hours by contrast tended to fare slightly worse (respectively 1.1 and 1.2 h differences for usual working-time in 2000 and 2015). In the case of part-timers for 2015, the difference was close to 2 h.

On the other hand, in only five out of nine cases did the mean working-time estimates derived from the calibrated LFS become statistically equivalent to those calculated with the work schedule. Welsh weighted *t* tests carried out on estimates from both sources gave non significant results (meaning that the mean did not significantly differ between the two datasets) for the usual hours of men working full-time time in 2015 and for women working full-time and part-time in 2015. Actual hours of both men and women working full-time also became equivalent. This was not the case however for women reporting part-time hours. For clarity purpose and also because they traditionally represent a smaller group, men working part-time were left out from Fig. 5. Calibration worked well for this group as calibrated estimates became equivalent to the work schedule in all year/instrument configurations. There are also indications that calibration worked best with 2015 data irrespective of the usual or actual instrument used. These results should be taken as indicative rather than definite proof as it would be wrong to assume that instruments in both surveys are completely equivalent. Cases where marked differences between *stylised* estimates are visible probably indicate that somehow the measurement are not perfectly identical which introduce noise in the comparison, for instance among men working full-time in 2000.

## Conclusion

This study has attempted to map the conceptual and empirical discrepancies between three types of instruments used for measuring weekly paid working time: stylised questions about usual and actual hours of work, and the weekly work schedule, a time diary instrument which records any 15 min long work episodes over a period of seven day. Using UK Labour Force Survey and Time Use Survey data from 2000 and 2015, it explored the gap between the latter—presumed to be more accurate by the literature—and the former two. Results showed that those working long hours tend to overestimate their working time, whilst those working short hours underestimate them. They also confirmed gender differences likely to overlap these given the prevalence of women in part-time jobs. Variations in daily working time were also found to be associated with the gap: respondents experiencing more volatile daily work routines over the week were likely to display a greater gap between stylised and work schedule recorded hours. Results also confirmed that instruments recording actual hours, that is hours worked the week prior to interview came closer to those estimated with time diary data than usual hours, which is likely to be due to the lesser cognitive burden involved. All in all by demonstrating the non random nature of the discrepancies between time diary and stylised instruments these results also further weaken the credibility of claims that these could simply represent the effect of a regression to the mean.

During a second stage, calibration weights were derived from OLS models of stylised working time in the UK TUS 2000 and 2015, then imputed to LFS data and results examined for full-time working working males as well as part-time and full-time working females. These markedly reduced the gap between stylised and work schedule estimates, especially in the case of actual hours of paid work. These results could pave the way to cost-effective improvements of traditional working-time estimates in mainstream social surveys. This could be implemented for instance by collecting work schedule data in social surveys such as the LFS at regular intervals and use it to calibrate working-time estimates for a number of subsequent issues of the data.

A number of questions remain unanswered by this study and should be addressed in future research. To start with, unobserved heterogeneity, especially among men who report more than 45 h of work per week need to be explored further as there are indications that these are the groups for which model-based calibration works worst. Another one would be to systematically compare statistical matching techniques used in this paper with more sophisticated ones relying on bayesian approach or bootstraping methods (Borra et al. 2013), whilst simultaneously exploring the consequences in terms of the gap between diary-recorded and stylised estimates. Finally, a systematic analysis of discrepancies between usual and actual stylised hours in future datasets could pave the way for a better understanding of the measurements errors associated with each one of these instruments.

## Footnotes

1 Note: in 2000 both work and full-time education were reported in the weekly work schedule. The number of respondents reporting paid work is therefore smaller than 6414.
